# Management of asymptomatic sporadic non-functioning pancreatic neuroendocrine neoplasms no larger than 2 cm: interim analysis of prospective ASPEN trial

**DOI:** 10.1093/bjs/znac267

**Published:** 2022-08-20

**Authors:** Stefano Partelli, Sara Massironi, Alessandro Zerbi, Patricia Niccoli, Wooil Kwon, Luca Landoni, Francesco Panzuto, Ales Tomazic, Alberto Bongiovanni, Gregory Kaltsas, Alain Sauvanet, Emilio Bertani, Vincenzo Mazzaferro, Martyn Caplin, Thomas Armstrong, Martin O Weickert, John Ramage, Eva Segelov, Giovanni Butturini, Stefan Staettner, Mauro Cives, Andrea Frilling, Carol Anne Moulton, Jin He, Florian Boesch, Andreas Selberheer, Orit Twito, Antonio Castaldi, Claudio G De Angelis, Sebastien Gaujoux, Katharina Holzer, Colin H Wilson, Hussein Almeamar, Emanuel Vigia, Francesca Muffatti, Martina Lucà, Andrea Lania, Jacques Ewald, Hongbeom Kim, Roberto Salvia, Maria Rinzivillo, Alojz Smid, Andrea Gardini, Marina Tsoli, Olivia Hentic, Samuele Colombo, Davide Citterio, Christos Toumpanakis, Emma Ramsey, Harpal S Randeva, Ray Srirajaskanthan, Daniel Croagh, Paolo Regi, Silvia Gasteiger, Pietro Invernizzi, Cristina Ridolfi, Marc Giovannini, Jin-Young Jang, Claudio Bassi, Massimo Falconi

**Affiliations:** School of Medicine, Vita-Salute San Raffaele University, Milan, Italy; Pancreas Translational and Clinical Research Centre, Pancreatic Surgery Unit, IRCCS San Raffaele Scientific Institute, Milan, Italy; Division of Gastroenterology and Centre for Autoimmune Liver Diseases, San Gerardo Hospital, Monza, Italy; Department of Medicine and Surgery, University of Milano-Bicocca, Monza, Italy; Department of Biomedical Sciences, Humanitas University, Pieve Emanuele, Milan, Italy; IRCCS Humanitas Research Hospital, Rozzano, Milan, Italy; Department of Medical Oncology, Paoli-Calmettes Institute, Marseille, France; Department of Surgery and Cancer Research Institute, Seoul National University College of Medicine, Seoul, Korea; General and Pancreatic Surgery Unit, Pancreas Institute, University of Verona Hospital Trust, Verona, Italy; Digestive Disease Unit, ENETS Centre of Excellence, Sant’ Andrea University Hospital, Rome, Italy; Department of Abdominal Surgery, University Medical Centre, Ljubijana, Slovenia; Osteoncology and Rare Tumours Centre (CDO-TR), IRCCS Istituto Romagnolo per lo Studio dei Tumori (IRST) ‘Dino Amadori’, Meldola, Italy; First Propaedeutic Department of Internal Medicine, National and Kapodistrian University of Athens, Athens, Greece; Department of Pancreatology, Hôpital Beaujon, University of Paris, Paris, France; Division of Gastrointestinal Surgery, IEO, European Institute of Oncology IRCCS, Milan, Italy; Gastrointestinal and Hepato-Pancreatic Surgery and Liver Transplantation Unit, Fondazione, IRCCS Istituto Nazionale Tumori (INT, National Cancer Institute) and Università degli Studi di Milano, Milan, Italy; ENETS Centre of Excellence, Neuroendocrine Tumour Unit, Royal Free Hospital, London, UK; Department of Hepatobiliary Surgery, Wessex NET Group ENETS Centre of Excellence, University Hospital Southampton, Southampton, UK; ARDEN NET Centre, ENETS Centre of Excellence, University Hospitals Coventry and Warwickshire NHS Trust and Warwick Medical School, University of Warwick, Coventry, UK; Kings Health Partners NET Centre, Kings College Hospital London, London, UK; Department of Oncology and Surgery (School of Clinical Sciences at Monash Health), Monash University, Clayton, Victoria, Australia; Department of Surgery, Pederzoli Hospital, Peschiera del Garda, Italy; Department of General, Visceral and Vascular Surgery, Salzkammergutklinikum Vöcklabruck, Vöcklabruck, Austria; Department of Biomedical Sciences and Human Oncology, University of Bari ‘Aldo Moro’, Bari, Italy; Department of Surgery and Cancer, Imperial College London, London, UK; Division of General Surgery, University of Toronto, Toronto, Ontario, Canada; Department of Surgery, University Health Network, Princess Margaret Cancer Centre, University of Toronto, Toronto, Ontario, Canada; Department of Surgery, Johns Hopkins University School of Medical, Baltimore, Maryland, USA; Department of General, Visceral, and Transplant Surgery, Ludwig-Maximilians-University Munich, Munich, Germany; Section Endocrine Surgery, Division of General Surgery, Department of Surgery, Medical University, Vienna, Austria; Endocrine Institute, Meir Medical Center, Kfar-Sava, Israel; Sackler Faculty of Medicine, Tel-Aviv University, Tel-Aviv, Israel; Department of Clinical Medicine and Surgery, University of Naples Federico II, Naples, Italy; Gastroenterology Unit, Department of Medical Sciences, City of Health and Science Hospital, Turin, Italy; Department of Digestive, Hepatobiliary and Endocrine Surgery, Paris Sorbonne University, Pitiè Salpétrière Hospital, Paris, France; Department of Visceral-, Thoracic- and Vascular Surgery, Section of Endocrine Surgery, University Hospital Marburg (UKGM), Marburg, Germany; Hepatopancreatobiliary and Transplant Unit, Freeman Hospital, Newcastle upon Tyne, UK; National NET Centre and ENETS Centre of Excellence, St Vincent’s University Hospital, Dublin, Ireland; Hepato-Biliary-Pancreatic and Transplantation Centre, Curry Cabral Hospital, CHULC, Lisbon, Portugal; School of Medicine, Vita-Salute San Raffaele University, Milan, Italy; Pancreas Translational and Clinical Research Centre, Pancreatic Surgery Unit, IRCCS San Raffaele Scientific Institute, Milan, Italy; Division of Gastroenterology and Centre for Autoimmune Liver Diseases, San Gerardo Hospital, Monza, Italy; Department of Medicine and Surgery, University of Milano-Bicocca, Monza, Italy; Department of Biomedical Sciences, Humanitas University, Pieve Emanuele, Milan, Italy; IRCCS Humanitas Research Hospital, Rozzano, Milan, Italy; Department of Medical Oncology, Paoli-Calmettes Institute, Marseille, France; Department of Surgery and Cancer Research Institute, Seoul National University College of Medicine, Seoul, Korea; General and Pancreatic Surgery Unit, Pancreas Institute, University of Verona Hospital Trust, Verona, Italy; Digestive Disease Unit, ENETS Centre of Excellence, Sant’ Andrea University Hospital, Rome, Italy; Department of Gastroenterology and Hepatology, University Medical Centre Ljubijana, Ljubljana, Slovenia; General and Oncological Surgery Unit, Morgagni-Pierantoni Hospital, Forlì, Italy; First Propaedeutic Department of Internal Medicine, National and Kapodistrian University of Athens, Athens, Greece; Department of Pancreatology, Hôpital Beaujon, University of Paris, Paris, France; Division of Gastrointestinal Surgery, IEO, European Institute of Oncology IRCCS, Milan, Italy; Gastrointestinal and Hepato-Pancreatic Surgery and Liver Transplantation Unit, Fondazione, IRCCS Istituto Nazionale Tumori (INT, National Cancer Institute) and Università degli Studi di Milano, Milan, Italy; ENETS Centre of Excellence, Neuroendocrine Tumour Unit, Royal Free Hospital, London, UK; Department of Hepatobiliary Surgery, Wessex NET Group ENETS Centre of Excellence, University Hospital Southampton, Southampton, UK; Warwick Medical School, University of Warwick, Coventry, UK; Kings Health Partners NET Centre, Kings College Hospital London, London, UK; Department of Oncology and Surgery (School of Clinical Sciences at Monash Health), Monash University, Clayton, Victoria, Australia; Department of Surgery, Pederzoli Hospital, Peschiera del Garda, Italy; Department of Visceral, Transplantation and Thoracic Surgery, Medical University of Innsbruck, Innsbruck, Austria; Division of Gastroenterology and Centre for Autoimmune Liver Diseases, San Gerardo Hospital, Monza, Italy; Department of Medicine and Surgery, University of Milano-Bicocca, Monza, Italy; Pancreatic Surgery Unit, Humanitas Clinical and Research Hospital—IRCCS, Rozzano, Milan, Italy; Department of Medical Oncology, Paoli-Calmettes Institute, Marseille, France; Department of Surgery and Cancer Research Institute, Seoul National University College of Medicine, Seoul, Korea; General and Pancreatic Surgery Unit, Pancreas Institute, University of Verona Hospital Trust, Verona, Italy; School of Medicine, Vita-Salute San Raffaele University, Milan, Italy; Pancreas Translational and Clinical Research Centre, Pancreatic Surgery Unit, IRCCS San Raffaele Scientific Institute, Milan, Italy

## Introduction

The incidence of non-functioning pancreatic neuroendocrine neoplasms (NF-PanNENs) has increased recently^1^. Traditionally, surgery has been the treatment of choice for localized NF-PanNENs, although evidence has emerged that active surveillance could be advocated for most asymptomatic tumours no larger than 2 cm^2–7^. However, the practice of active surveillance varies considerably and, contrary to current recommendations^8–10^, many patients still undergo surgical resection^11–13^.

Current evidence is limited by the retrospective design of studies and the small number of patients. The present study is the most extensive prospective investigation to date on small, asymptomatic NF-PanNENs. The aim was to define the optimal management of incidentally found, sporadic NF-PanNENs no larger than 2 cm.

## Methods

This was a prospective, non-randomized, international, multicentre, cohort study (NCT03084770). This report describes the results of the prespecified interim analysis. Overall, 41 centres have been included. The study protocol was published previously^14^ (*Appendix S1*). Briefly, CT or MRI was mandatory for all patients. The diagnosis must have been proven by a positive fine-needle aspiration (biopsy) (FNA(B)) or positive ^68^Ga-labelled DOTA PET. The treatment—active surveillance or surgical resection—was decided by the referring centre. Because current guidelines^8–10^ suggest surveillance for asymptomatic NF-PanNENs 2 cm or smaller in size, treating physicians were asked to indicate the reason for choosing surgery. An aggressive feature was defined by one or more of the following features: Ki-67 over 20 per cent, perineural invasion, microvascular invasion, nodal metastases, or distant metastases.

## Results

**Fig. 1:**
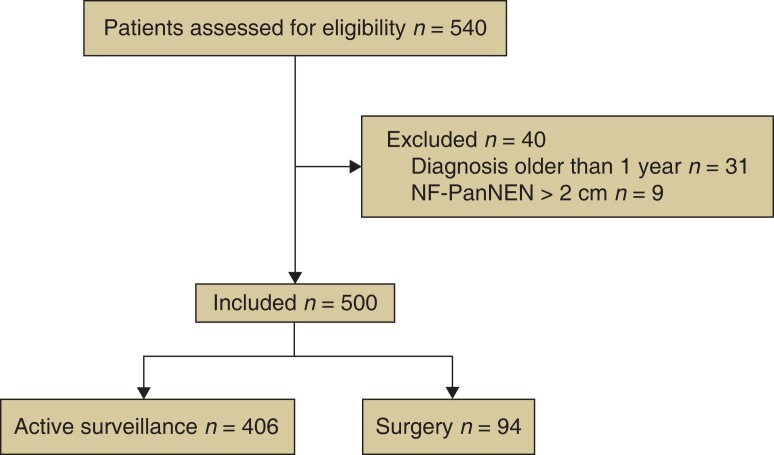
Study flow chart NF-PanNEN, non-functioning pancreatic neuroendocrine neoplasm.

The study flow diagram is shown in *Fig. 1*. After initial screening, all the patients had at least positive ^68^Ga-labelled DOTA PET and/or a positive FNA(B) for NF-PanNEN.

*Table 1* summarizes demographics and clinical characteristics by the type of management. Younger age, larger tumour size, lower BMI, dilated main pancreatic duct (MPD), and enrolment of the patient in a surgical centre were associated more frequently with surgery. Global quality of life at diagnosis was similar in the two groups (*Fig. S1*). Overall, distant metastases were present in 4 patients (0.08 per cent), all of whom underwent surgery. On multivariable analysis, factors associated with surgery were: age 64 years or less (OR 2.5; *P* < 0.001), radiological size larger than 10 mm (OR 1.9; *P* = 0.030), MPD: over 3 mm (OR 3.4; *P* < 0.001), surgical centre (OR 2.0; *P* = 0.012), and Hospital Anxiety and Depression Scale—anxiety score above 3 and no more than 6 (OR 2.0; *P* = 0.029) (*Table S1*). Indication for surgery was attributed to patient’s preference in 42 instances (45 per cent), centre’s preference in 37 (39 per cent), MPD dilatation in 11 (12 per cent), and distant metastases in 4 (4 per cent).

**Table 1:** Characteristics of patients in ASPEN study

	Overall (*n* = 500)	Active surveillance (*n* = 406)	Surgical resection (*n* = 94)	*P*§
**Sex**				0.529
F	238 (47.6)	196 (48.3)	42 (45)	
M	262 (52.4)	210 (51.7)	52 (55)	
**Age (years), median (i.q.r.)**	64 (54–71)	65 (56–71)	59 (51–68)	<0.001¶
**BMI (kg/m^2^)**				0.052
≤ 25	175 (35.0)	134 (33.0)	41 (44)	
> 25	325 (65.0)	272 (67.0)	53 (56)	
**Diabetes**				0.268
No	417 (83.4)	335 (82.5)	82 (87)	
Yes	83 (16.6)	71 (17.5)	12 (13)	
**ECOG PS score**				0.305
0	436 (87.2)	349 (86.0)	87 (93)	
1	52 (10.4)	45 (11.1)	7 (7)	
≥ 2	12 (2.4)	12 (2.9)	0 (0)	
**Year of diagnosis**				0.059
2017–2018	271 (54.2)	212 (52.2)	59 (63)	
2019–2020	229 (45.8)	194 (47.8)	35 (37)	
**Site of lesion**				0.323
Head	121 (24.2)	101 (24.9)	20 (21)	
Uncinate process	55 (11.0)	48 (11.8)	7 (8)	
Body	167 (33.4)	136 (33.5)	31 (33)	
Tail	157 (31.4)	121 (29.8)	36 (38)	
Radiological tumour size (mm), mean(s.d.)*	13.2 (4.1)	12.9 (3.9)	14.8 (4.5)	<0.010#
**rN status**				1.000
rN0	499 (99.8)	406 (100)	93 (99)	
rN1	1 (0.02)	0 (0)	1 (1)	
**rM status**				0.671
rM0	496 (99.2)	406 (100)	90 (96)	
rM1	4 (0.08)	0 (0)	4 (4)	
**MPD diameter (mm), mean(s.d.)**	2.9 (3.4)	2.5 (3.1)	4.3 (4.0)	0.001#
**CgA (ng/ml), median (i.q.r.)**	61 (30–36)	60 (25–106)	63 (44–123)	0.259¶
**[^18^F]FDG PET**				0.454
Not performed	427 (85.4)	344 (84.7)	83 (88)	
Negative	55 (11.0)	48 (11.8)	7 (8)	
Positive	18 (3.6)	14 (3.4)	4 (4)	
**FNA(B)**				0.001
Not performed	170 (34.0)	124 (30.5)	46 (49)	
Negative	45 (9.0)	34 (8.4)	11 (12)	
Positive	285 (57.0)	248 (61.1)	37 (39)	
**Tumour grade†**				<0.001
PanNET-G1	195 (68.4)	179 (72.2)	16 (43)	
PanNET-G2	22 (7.7)	12 (4.8)	10 (27)	
Not evaluable	68 (23.9)	57 (23.0)	11 (30)	
**Surgical centre**				0.006
No	178 (35.6)	156 (38.4)	22 (23)	
Yes	322 (64.4)	250 (61.6)	72 (77)	
**HADS score‡**				0.295
≤ 5	167 (33.4)	142 (35.0)	25 (27)	
6–12	172 (34.4)	137 (33.7)	35 (37)	
> 12	161 (32.2)	127 (31.3)	34 (36)	
**HADS—anxiety score‡**				0.108
≤ 3	148 (29.6)	126 (31.0)	22 (23)	
4–6	178 (35.6)	136 (33.5)	42 (45)	
> 6	174 (34.8)	144 (35.5)	30 (32)	
**HADS—depression score‡**				
≤ 2	114 (22.8)	95 (23.4)	19 (20)	0.563
3–4	207 (41.4)	170 (41.9)	37 (39)	
> 4	179 (35.8)	141 (34.7)	38 (41)	

Values are *n* (%) unless otherwise indicated. *Maximum size on radiological imaging or endoscopic ultrasonography. †Evaluated for patients with positive fine-needle aspiration (biopsy) (FNA(B) specimen. ‡Categorized by tertiles of Hospital Anxiety and Depression Scale (HADS) distribution. ECOG PS, Eastern Cooperative Oncology Group performance status; MPD, main pancreatic duct; CgA, chromogranin A; FDG, fluorodeoxyglucose. §Pearson χ^2^ test, except ¶Wilcoxon Mann–Whitney test and #*t* test.

Surgical outcomes are summarized in *Table S2*. Minimally invasive, either laparoscopic or robot-assisted, was the preferred approach in 55 per cent of patients. Severe complications (defined as those with a Clavien–Dindo grade^15^ of more than III) occurred in 13 per cent of patients whereas the mortality rate was zero. Final pathological examination characteristics are listed in *Table S3*. The choice of standard pancreatectomy over an atypical resection was justified by the need to perform an adequate lymphadenectomy in 52 patients (54 per cent) and the proximity of the nodule to the MPD in 23 (25 per cent). One or more aggressive histological features were observed in 19 patients (20 per cent). Of these 19 patients, 17 had a radiological tumour size larger than 10 mm. The remaining 2 patients with radiological tumour size less than 10 mm had a dilated MPD on preoperative imaging. In 5 of the 19 patients with aggressive features, the radiological MPD was larger than 3 mm.

After a median follow-up of 25 (i.q.r. 16–35) months, all patients were alive apart from 3 who died from causes unrelated to NF-PanNENs. Only 1 patient in the surgical group, who had liver metastases at diagnosis, eventually developed liver recurrence.

In the surveillance group, 9 patients (2 per cent) underwent surgery during follow-up. The reason for surgery was increasing tumour size in 4 patients, increased MPD dilatation in 3, and patient’s preference in 2.

## Discussion

A non-operative strategy seems safe as only a negligible fraction of patients had an increase in tumour size and no patient developed distant metastases during follow-up. These results are consistent with the preliminary findings of a recent prospective study^6^, although the present series included a five-fold larger number of patients and compared the two types of management of asymptomatic small NF-PanNENs, leaving the therapeutic decision (surveillance *versus* surgery) to the treating centres.

Other factors that contributed to the decision to resect a NF-PanNEN of 2 cm or smaller were younger age, tumour size over 1 cm, and the presence of MPD dilatation. Furthermore, patient’s preference was the main reason for choosing surgery in many instances. This attitude might be explained by patients’ anxiety and by the ongoing debate in the scientific community about the optimal management of these lesions. Moreover, the current guidelines^8–10^ suggest that surveillance is recommended, especially for older patients, and this may explain why young age was an important factor in deciding on a surgical approach more frequently. In the present experience, it was found that nearly 20 per cent of resected tumours had one or more aggressive features. Notably, nearly all the lesions that presented at least one aggressive feature were also larger than 1 cm.

The optimal cut-off for considering NF-PanNENs as low-risk lesions is a matter of ongoing controversy. The European Neuroendocrine Tumor Society^8^ and National Comprehensive Cancer Network^10^ guidelines consider observation for lesions no larger than 2 cm. On the other hand, North American Neuroendocrine Tumor Society^9^ guidelines suggest that the treatment of asymptomatic NF-PanNENs between 1 and 2 cm in size should be individualized. The present findings seem to support these latter recommendations. The presence of MPD dilatation should be promptly recognized and always considered as a major sign of concern because of the strong correlation with aggressive features, as described previously^16^. Another possible role in predicting the biological behaviour of these small nodules may be played by novel promising biomarkers such as NETest^17^. Finally, another important result was the detection of synchronous liver metastases in four patients, which demonstrates a real, although rare, potential for distant spread also among NF-PanNENs of 2 cm or smaller.

In conclusion, active surveillance is the preferred approach for sporadic, asymptomatic, NF-PanNENs no larger than 2 cm. An active surveillance strategy seems safe, but the measurable risk of distant metastases, as well as the presence of histological characteristics of aggressiveness in almost one-fifth of operated tumours, necessitates personalized management for lesions larger than 1 cm as well as for young patients and in the presence of measurable growth of the nodule. Moreover, surgery is always mandatory for small NF-PanNENs with a dilated MPD. According to the protocol, the study will be concluded 1 year after the enrolment of the last patient. Nevertheless, as these preliminary results showed only a very low rate of patients with tumour growth after a median follow-up of 2 years, longer follow-up is probably needed for definitive conclusions to be reached.

## Supplementary Material

znac267_Supplementary_Data

## References

[1] Dasari A , Shen C, Halperin D, Zhao B, Zhou S, Xu Y et al Trends in the incidence, prevalence, and survival outcomes in patients with neuroendocrine tumors in the United States. JAMA Oncol 2017;3:1335–1342 28448665 10.1001/jamaoncol.2017.0589 PMC5824320

[2] Lee LC , Grant CS, Salomao DR, Fletcher JG, Takahashi N, Fidler JL et al Small, nonfunctioning, asymptomatic pancreatic neuroendocrine tumors (PNETs): role for nonoperative management. Surgery 2012;152:965–974 23102679 10.1016/j.surg.2012.08.038

[3] Sadot E , Reidy-Lagunes DL, Tang LH, Do RKG, Gonen M, D’Angelica MI et al Observation *versus* resection for small asymptomatic pancreatic neuroendocrine tumors: a matched case–control study. Ann Surg Oncol 2016;23:1361–1370 26597365 10.1245/s10434-015-4986-1 PMC4798427

[4] Barenboim A , Lahat G, Nachmany I, Nakache R, Goykhman Y, Geva R et al Resection *versus* observation of small asymptomatic nonfunctioning pancreatic neuroendocrine tumors. J Gastrointest Surg 2019;24:1366–1374 31197692 10.1007/s11605-019-04285-y

[5] Partelli S , Cirocchi R, Crippa S, Cardinali L, Fendrich V, Bartsch DK et al Systematic review of active surveillance *versus* surgical management of asymptomatic small non-functioning pancreatic neuroendocrine neoplasms. Br J Surg 2017;104:34–41 27706803 10.1002/bjs.10312

[6] Heidsma CM , Engelsman AF, Van Dieren S, Stommel MWJ, de Hingh I, Vriens M et al Watchful waiting for small non-functional pancreatic neuroendocrine tumours: nationwide prospective cohort study (PANDORA). Br J Surg 2021;108:888–891 33783475 10.1093/bjs/znab088 PMC10364894

[7] Bettini R , Partelli S, Boninsegna L, Capelli P, Crippa S, Pederzoli P et al Tumor size correlates with malignancy in nonfunctioning pancreatic endocrine tumor. Surgery 2011;150:75–82 21683859 10.1016/j.surg.2011.02.022

[8] Partelli S , Bartsch DK, Capdevila J, Chen J, Knigge U, Niederle B et al ENETS consensus guidelines for the standards of care in neuroendocrine tumours: surgery for small intestinal and pancreatic neuroendocrine tumours. Neuroendocrinology 2017;105:255–265 28237989 10.1159/000464292

[9] Howe JR , Merchant NB, Conrad C, Keutgen XM, Hallet J, Drebin JA et al The North American Neuroendocrine Tumor Society consensus paper on the surgical management of pancreatic neuroendocrine tumors. Pancreas 2020;49:1–33 31856076 10.1097/MPA.0000000000001454 PMC7029300

[10] Shah MH , Goldner WS, Halfdanarson TR, Bergsland E, Berlin JD, Halperin D et al NCCN guidelines insights: neuroendocrine and adrenal tumors, version 2.2018. J Natl Compr Canc Netw 2018;16:693–702 29891520 10.6004/jnccn.2018.0056

[11] Partelli S , Mazza M, Andreasi V, Muffatti F, Crippa S, Tamburrino D et al Management of small asymptomatic nonfunctioning pancreatic neuroendocrine tumors: limitations to apply guidelines into real life. Surgery 2019;166:157–163 31109657 10.1016/j.surg.2019.04.003

[12] Mintziras I , Keck T, Werner J, Fichtner-Feigl S, Wittel U, Senninger N et al Implementation of current ENETS guidelines for surgery of small (≤ 2 cm) pancreatic neuroendocrine neoplasms in the German Surgical Community: an analysis of the prospective DGAV StuDoQ|Pancreas Registry. World J Surg 2018;43:175–182 10.1007/s00268-018-4751-2 30097704

[13] Chivukula SV , Tierney JF, Hertl M, Poirier J, Keutgen XM. Operative resection in early stage pancreatic neuroendocrine tumors in the United States: are we over- or undertreating patients? Surgery 2020;167:180–186 31537303 10.1016/j.surg.2019.04.061

[14] Partelli S , Ramage JK, Massironi S, Zerbi A, Kim HB, Niccoli P et al Management of asymptomatic sporadic nonfunctioning pancreatic neuroendocrine neoplasms (ASPEN) ≤ 2 cm: study protocol for a prospective observational study. Front Med 2020;7:1–8 10.3389/fmed.2020.598438 PMC7785972 33425946

[15] Dindo D , Demartines N, Clavien PA. Classification of surgical complications: a new proposal with evaluation in a cohort of 6336 patients and results of a survey. Ann Surg 2004;240:205–213 15273542 10.1097/01.sla.0000133083.54934.ae PMC1360123

[16] Zhou B , Zhan C, Xiang J, Ding Y, Yan S. Clinical significance of the preoperative main pancreatic duct dilation and neutrophil-to-lymphocyte ratio in pancreatic neuroendocrine tumors (PNETs) of the head after curative resection. BMC Endocr Disord 2019;19:123 31718651 10.1186/s12902-019-0454-4 PMC6852769

[17] Partelli S , Andreasi V, Muffatti F, Schiavo Lena M, Falconi M. Circulating neuroendocrine gene transcripts (NETest): a postoperative strategy for early identification of the efficacy of radical surgery for pancreatic neuroendocrine tumors. Ann Surg Oncol 2020;27:3928–3936 32253675 10.1245/s10434-020-08425-6

