# Supplementary material for: Management of asymptomatic sporadic non-functioning pancreatic neuroendocrine neoplasms no larger than 2 cm: interim analysis of prospective ASPEN trial
Source: Br J Surg. 2022 Aug 20;109(12):1186–90. doi: 10.1093/bjs/znac267 (PMC10364756; doi:10.1093/bjs/znac267)
Supplement: znac267_Supplementary_Data [file znac267_supplementary_data.zip › Supplementary_Appendix_1.docx]

**A Prospective Evaluation of the Management of Sporadic Asymptomatic Nonfunctioning Pancreatic Neuroendocrine Neoplasms ≤ 2 cm**

**Short Title:**

**Asymptomatic Small Pancreatic Endocrine Neoplasms (ASPEN study)**

Protocol Version No: 1.0 – 08 Nov 2016

**Study Chief Investigator:** Professor Massimo Falconi

**CONTENTS**

1. Study Summary……………………………………………………………………………..3
2. Background………………………………………………………………………………….4
3. Aim……………………………………………………………………………………………5
4. Study Design………………………………………………………………………………..5
5. Study Population………………………………………………………………………….5-6
   1. Population……………………………………………………………………………….5
   2. Inclusion criteria…………………………………………………………………………6
   3. Exclusion criteria………………………………………………………………………..6
   4. Sample Size Calculation……………………………………………………………….6
6. Treatment of Subjects………………………………………………………………………7
   1. Diagnostic work-up before inclusion………………………………………………….7
   2. NF-PNEN management ………………………………………………….7
   3. Data collection…………………………………………………………………………..8
7. Methods………………………………………………………………………………………9
   1. Study Endpoints………………………………………………………………………...9
8. Statistical Analysis………………………………………………………………………….10
9. Authorship Policy……………………………………………………………………………11
10. References……………………………………………………………………………....11-13

Appendix 1-Study Flowchart………………………………………………………………………14

Appendix 2-Scheme of active surveillance ……………………………………………..……….15

Appendix 3- Scheme after surgical resection…………………………………………………….16

Appendix 4-HADS questionnaire……………………………………………………………….….17

Appendix 5- EORTC QLQ-C30 Questionnaire (version 3.0)………………………………..18-19

Appendix 6 - EORTC QLQ – GI.NET21…………………………………………………………..20

# STUDY SUMMARY

| **Chief Investigator** | **Prof Massimo Falconi**  Head of Pancreatic Surgery  IRCCS Ospedale San Raffaele  Via Olgettina 60 – 20132 Milan – Italy  Telephone: +39 0226436046  Email: [falconi.massimo@hsr.it](mailto:falconi.massimo@hsr.it) |
| --- | --- |
| **Study Coordinator** | **Dr Stefano Partelli**  Pancreatic Surgery Division  IRCCS Ospedale San Raffaele  Via Olgettina 60 – 20132 Milan – Italy  Telephone: +39 0226437697  Email: [partelli.stefano@hsr.it](mailto:partelli.stefano@hsr.it)  **Dr ssa Francesca Muffatti**  Pancreatic Surgery division  IRCCS Ospdele San Raffaele  Via Olgettina 60 – 20132 Milan – Italy  Telephone: 0226437811  Email: [francesca.muffatti@hsr.it](mailto:francesca.muffatti@hsr.it) |
| **Study Title** | **A Prospective Evaluation of the Management of Sporadic Asymptomatic Nonfunctioning Pancreatic Neuroendocrine Neoplasms ≤ 2 cm** |
| **Short title** | **Asymptomatic Small Pancreatic Endocrine Neoplasms (ASPEN study)** |
| **Study Design** | Prospective international multicentre cohort study |
| **Study Patients** | **Inclusion criteria**  • Age > 18 years  • Individuals with asymptomatic sporadic NF-PNEN ≤ 2 cm  • Diagnosis has to be proven by a positive fine-needle aspiration (FNA) or by the presence of a measurable nodule on high-quality imaging techniques that is positive at 68Gallium DOTATOC-PET scan or Octreoscan.  • Patients who undergo surgery for NF-PNEN<2cm within 12 months. In these cases, diagnosis has to be proven by histological confirmation of NF-PNEN  • Informed consent  **Exclusion criteria**  **•** NF-PNEN > 2 cm of maximum diameter  • Presence of genetic syndrome (MEN1, VHL, NF)  • Presence of symptoms (specific symptoms suspicious of a clinical syndrome related to hypersecretion of bioactive compounds or unspecific symptoms) |
| **Research Aim** | To evaluate the most appropriate management of sporadic asymptomatic non-functioning pancreatic neuroendocrine neoplasms (NF-PNEN) ≤ 2 cm |

# BACKGROUND

Nonfunctioning pancreatic neuroendocrine neoplasms (NF-PNEN) are rare tumors that exhibit a wide heterogeneity of aggressiveness. The current World Health Organization (WHO) classification identified three categories of NF-PNEN (NF-PNEN-G1, NF-PNEN-G2, and NF-PNEC-G3) based on Ki-67 value [^1^](#_ENREF_1). Indications for surgery include the presence of a localized NF-PNEN in the absence of distant metastases as curative resection of these tumors is associated with favourable prognosis especially for low grade [^2-4^](#_ENREF_2).

In the last decade a dramatic increase in diagnosis of small, incidentally discovered, NF-PNEN was observed[^5^](#_ENREF_5) [^6^](#_ENREF_6). Several studies highlighted the role of incidental diagnosis as a powerful prognostic factor for NF-PNEN[^7^](#_ENREF_7) [^8^](#_ENREF_8). Moreover, other investigators observed a clear relationship between the tumor diameter and low risk of malignancy and systemic progression.[^9-11^](#_ENREF_9).

In particular, a tumor size ≤ 2 cm seems to be associated with a negligible risk of disease recurrence and with a very low incidence of aggressive features such as lymph node involvement[^4^](#_ENREF_4) [^12^](#_ENREF_12).

On this basis, the European Neuroendocrine Tumor Society (ENETS) proposed a “wait and see” approach for small NF-PNEN when incidentally discovered[^13^](#_ENREF_13). Since then, various series evaluated the safety of a conservative management for small, sporadic, incidentally diagnosed, NF-PNEN[^14-19^](#_ENREF_14).

After a median follow-up of 28-45 months, all the studies confirmed that an intensive surveillance for incidental and small NF-PNEN is safe in selected cases. None of the patients in the observational group deceased for disease or had disease progression when lesions were less than 2 cm. Nevertheless, available data are based only on retrospective series with a significant heterogeneity of inclusion criteria and different tumor diameter cut-off^14-18^ and the appropriate management of this entities (surveillance versus surgery) is still a matter of debate.

# AIM

To evaluate the most appropriate management of sporadic asymptomatic NF-PNEN ≤ 2 cm

# STUDY DESIGN

The study is designed as a prospective international (including also non-European institutions) multicentre cohort study, which will be coordinated by the Pancreatic Surgery Unit of San Raffaele Scientific Institute (Lead Study Centre) under the auspices of the European Neuroendocrine Tumor Society (ENETS).

The study duration is 6 years, patients will be recruited for 5 years from December 2016 to December 2021, with a follow up of 1 year at least (end of the study: December 2022). After 2 years of recruitment an interim analysis will be performed in December 2018. San Raffaele Scientific Institute will be the lead centre from where the international study will be managed and coordinated. Participating study centre will identify, recruit patients and will send pseudoanonimised data of patients to the lead centre, which is responsible for statistical analysis, storing and controlling data. The research database will be managed and analysed by the Lead Study centre research team. Study participation is voluntary, there will be no reimbursement for patients.

# STUDY POPULATION

## 5.1 Population

This study enrols individuals with sporadic incidentally discovered NF-PNEN ≤ 2 cm.

## 5.2 Inclusion Criteria

- Age > 18 years
- Individuals with asymptomatic sporadic NF-PNEN ≤ 2 cm
- Diagnosis has to be proven by a positive fine-needle aspiration (FNA) or by the presence of a measurable nodule on high-quality imaging technique (CT or MR) that is positive at ^68^Gallium DOTATOC-PET scan or Octreoscan.
- Patients who undergo surgery for NF-PNEN<2cm within 12 months. In these cases, diagnosis has to be proven by histological confirmation of NF-PNEN
- Informed consent

**5.3 Exclusion Criteria**

- NF-PNEN > 2 cm of maximum diameter
- Presence of genetic syndrome (MEN1, VHL, NF)
- Presence of symptoms (specific symptoms suspicious of a clinical syndrome related to hypersecretion of bioactive compounds) or unspecific symptoms

**5.4 Sample Size Calculation**

Based on a reported incidence rate of PNEN of 0.4/100.000 inhabitants[^5^](#_ENREF_5) [^20^](#_ENREF_20) and considering a rate of PNEN<2cm of 20%, we estimate an incidence of around 580 PNEN<2cm per year only in Europe. Worldwide the estimation of new PNEN<2 cm is around 29840 cases in five years. In the study period we aim to include at least 1000 patients during the study period.

# TREATMENT OF SUBJECTS

Study flowchart is provided in **Appendix 1**.

## 6.1 Diagnostic work-up before inclusion

The goal of the diagnostic work-up is to characterize the neoplasm and to rule out other lesions (i.e. ductal adenocarcinoma, accessory spleen, solid serous cystadenoma). This work-up should have taken place no more than 12 months prior to inclusion. A high quality cross-sectional imaging study, either Computed Tomography (CT) or Magnetic Resonance Imaging (MRI) is mandatory. Diagnosis has to be proven by a positive fine-needle aspiration (FNA) or by the presence of a measurable nodule on high-quality imaging technique (CT or MR) that is positive at 68Gallium DOTATOC-PET scan or Octreoscan. Patients who undergo surgery for NF-PNEN<2cm within 12 months can also be enrolled. In these cases, diagnosis has to be proven by histological confirmation of NF-PNEN. CgA should be determined at treating physician’s discretion.

**6.2 NF-PNEN management**

NF-PNEN management will be decided at the hospital and all therapeutics decision will be decided/coordinated by the treating physician.

Advised surveillance strategy consists of imaging studies (MR or EUS or US), every 6 months for the first two years and yearly thereafter for five years in the absence of significant changes on imaging or symptoms appearance. During surveillance, a high-quality imaging technique (MRI or CT) is mandatory at least every 12 months.

Determination of CgA during follow-up is at physician’s discretion. During follow-up, the treating physician is responsible for patient management and decision-making. If follow-up parameters change during follow-up, the decision for a more diagnostic work-up, surgery, or an intensified follow-up schedule is at the discretion of the treating physician (**Appendix 2**). If surgical resection is warranted, timing and type of resection will be established by the treating physician. Suggested scheme of follow up after surgery is depicted in **Appendix 3.**

Date of surgery does not change the timing of follow up which starts from the date of enrolment. If during surveillance a NF-PNEN size increases > 2 cm and surgery is not performed, the reason should be stated. In this case patient is not excluded and follow-up will continue regularly.

**6.3 Data collection**

Treating physicians will be asked to fill case record forms (CRF). The following CRF will be provided:

- CRF 1: Diagnostic work-up before inclusion
- CRF 2: Active surveillance outcomes
- CRF 3: Surgical outcomes
- CRF 4: Pathological findings
- CRF 5: Follow-up after surgery

Patients will be asked to fill a questionnaire regarding the burden of NF-PNEN (Hospital Anxiety and Depression Scale – HADS) and two questionnaires regarding quality of life of patients with NF-PNEN (EORTC QLQ-C30 - version 3.0 and EORTC QLQ-GI.NET21). All three module will be administered at initial diagnosis, during surveillance and during follow-up after surgery at each visit.[^21^](#_ENREF_21) [^22^](#_ENREF_22) **(Appendix 4, Appendix 5, Appendix 6).**

Data will be recorded by the treating physician on a specific web-based site. Patients demographics will be not included in the CRF, and every patient will have an unique code, assigned by the treating centre, that will identify the Centre and the patient itself (e.g. SR001 San Raffaele Hospital patient number 1).

# METHODS

## Study Endpoints

### Main study endpoint

### The primary endpoint is disease/progression-free survival, defined as the time from study enrolment to the first evidence of progression (surveillance group) or recurrence of disease (surgery group) or death from disease.

### Secondary study endpoints

### To evaluate the frequency of asymptomatic sporadic NF-PNEN ≤ 2 cm among overall sporadic NF-PNEN. Participating centers are required to give yearly the number of patients with NF-PNEN referred to their institution.

### The outcome of patients with an indication for NF-PNEN resection, in terms of the number of operated patients, surgical procedures, morbidity, mortality, and NF-PNEN recurrence.

### NF-PNEN evolution, in terms of development of symptoms, tumour growth, development of distant metastases and secondary pancreatic duct dilatation.

### The perceived burden of surveillance or follow-up after surgery for participants, as assessed by questionnaires regarding attitude towards surveillance and general anxiety and depression (Hospital Anxiety and Depression scale, HADS[^22^](#_ENREF_22)). Moreover we ask patients to fill out EORTC QLQ-C30 (version 3) and EORTC QLQ-GI.NET21 Module.

# STATISTICAL ANALYSIS

The first analyses will regard the data collected within the first 2 years. This report will contain purely descriptive statistics, according to the study endpoints described. The second analysis, after 5 years, will give an update of the first report and provide a more in depth analysis of the primary and secondary study endpoints.

Baseline patient and NF-PNEN characteristics will be described. Also, descriptive of the primary and secondary endpoints will be given. Depending on distributional properties of the observed variable, percentages, means ± standard deviation (SD), or medians with interquartile ranges (IQR) will be reported. Statistical significance will be assessed with use of the Student’s t-test for normally distributed continuous data; either the chi-square test for categorical data (with Yates’ correction when appropriate) or Fisher exact test for categorical data; and the median test for non-normally distributed continuous data. All reported p-values will be two-sided and a value < 0.05 will be considered to be significant. Data will be analysed with SPSS (SPSS Inc, Chicago, Illinois). For the primary endpoints, univariate comparisons will be conducted, to identify individual patient and NF-PNEN risk factors for progression/recurrence. As primary potential risk factors are considered; 1. NF-PNEN size at initial diagnosis, 2. NF-PNEN growth during follow-up, 3. serum CgA, 4.. Patient age, 5. Ki67.

Outcomes will be evaluated in the intention-to-treat population on the basis of treating physician-assessed tumor progression/recurrence. Survival analysis techniques and Cox regression with time-dependent recurrent covariates measures will be applied. Progression/recurrence is defined according to Response Evaluation Criteria in Solid Tumors (RECIST)[^23^](#_ENREF_23) version 1.0 criteria. In the surveillance group progression is defined as the appearance of distant metastases and/or local signs of invasiveness (I.e. vascular or nearby organs invasion). The mere tumor size increasing will be not considered a sign of progression unless it reaches > 2 cm of maximum diameter. The two groups of patients (active surveillance and surgical resection group) will not be directly compared. Rate of expect events is 0-10% for the two groups^19^ .

Multivariate survival analysis will only be performed if the number of events will be > 30. The potential risk factors, given above, will have first interest.

# AUTHORSHIP POLICY

The first, senior author and corresponding author will be from the Lead Centre.

For each participating centre the number of authors will depend on the number of patients recruited. One author will be considered between 5 and 10 patients included, 2 authors between 10 and 20 patients included, 3 authors for more than 20. For those centres that will include less than 5 patients, the responsible of the centre will be included in the acknowledgement.

# REFERENCES

1. Bosman FT CF, Hruban RH, Theise ND. WHO classification of tumors of the digestive system. International Agency for Research on Cancer (IARC). Lyon, 2010.

2. Falconi M, Eriksson B, Kaltsas G, Bartsch DK, Capdevila J, Caplin M, et al. ENETS Consensus Guidelines Update for the Management of Patients with Functional Pancreatic Neuroendocrine Tumors and Non-Functional Pancreatic Neuroendocrine Tumors. Neuroendocrinology 2016;103(2):153-71.

3. Jilesen AP, van Eijck CH, Busch OR, van Gulik TM, Gouma DJ, van Dijkum EJ. Postoperative Outcomes of Enucleation and Standard Resections in Patients with a Pancreatic Neuroendocrine Tumor. World J Surg 2015.

4. Hashim YM, Trinkaus KM, Linehan DC, Strasberg SS, Fields RC, Cao D, et al. Regional lymphadenectomy is indicated in the surgical treatment of pancreatic neuroendocrine tumors (PNETs). Ann Surg 2014;259(2):197-203.

5. Kuo EJ, Salem RR. Population-level analysis of pancreatic neuroendocrine tumors 2 cm or less in size. Ann Surg Oncol 2013;20(9):2815-21.

6. Vagefi PA, Razo O, Deshpande V, McGrath DJ, Lauwers GY, Thayer SP, et al. Evolving patterns in the detection and outcomes of pancreatic neuroendocrine neoplasms: the Massachusetts General Hospital experience from 1977 to 2005. Arch Surg 2007;142(4):347-54.

7. Crippa S, Partelli S, Zamboni G, Scarpa A, Tamburrino D, Bassi C, et al. Incidental diagnosis as prognostic factor in different tumor stages of nonfunctioning pancreatic endocrine tumors. Surgery 2014;155(1):145-53.

8. Birnbaum DJ, Gaujoux S, Cherif R, Dokmak S, Fuks D, Couvelard A, et al. Sporadic nonfunctioning pancreatic neuroendocrine tumors: prognostic significance of incidental diagnosis. Surgery 2014;155(1):13-21.

9. Bettini R, Partelli S, Boninsegna L, Capelli P, Crippa S, Pederzoli P, et al. Tumor size correlates with malignancy in nonfunctioning pancreatic endocrine tumor. Surgery 2011;150(1):75-82.

10. Haynes AB, Deshpande V, Ingkakul T, Vagefi PA, Szymonifka J, Thayer SP, et al. Implications of incidentally discovered, nonfunctioning pancreatic endocrine tumors: short-term and long-term patient outcomes. Arch Surg 2011;146(5):534-8.

11. Cherenfant J, Stocker SJ, Gage MK, Du H, Thurow TA, Odeleye M, et al. Predicting aggressive behavior in nonfunctioning pancreatic neuroendocrine tumors. Surgery 2013;154(4):785-91; discussion 91-3.

12. Partelli S, Gaujoux S, Boninsegna L, Cherif R, Crippa S, Couvelard A, et al. Pattern and clinical predictors of lymph node involvement in nonfunctioning pancreatic neuroendocrine tumors (NF-PanNETs). JAMA Surg 2013;148(10):932-9.

13. Falconi M, Bartsch DK, Eriksson B, Kloppel G, Lopes JM, O'Connor JM, et al. ENETS Consensus Guidelines for the management of patients with digestive neuroendocrine neoplasms of the digestive system: well-differentiated pancreatic non-functioning tumors. Neuroendocrinology 2012;95(2):120-34.

14. Gaujoux S, Partelli S, Maire F, D'Onofrio M, Larroque B, Tamburrino D, et al. Observational study of natural history of small sporadic nonfunctioning pancreatic neuroendocrine tumors. J Clin Endocrinol Metab 2013;98(12):4784-9.

15. Lee LC, Grant CS, Salomao DR, Fletcher JG, Takahashi N, Fidler JL, et al. Small, nonfunctioning, asymptomatic pancreatic neuroendocrine tumors (PNETs): role for nonoperative management. Surgery 2012;152(6):965-74.

16. Jung JG, Lee KT, Woo YS, Lee JK, Lee KH, Jang KT, et al. Behavior of Small, Asymptomatic, Nonfunctioning Pancreatic Neuroendocrine Tumors (NF-PNETs). Medicine (Baltimore) 2015;94(26):e983.

17. Sadot E, Reidy-Lagunes DL, Tang LH, Do RK, Gonen M, D'Angelica MI, et al. Observation versus Resection for Small Asymptomatic Pancreatic Neuroendocrine Tumors: A Matched Case-Control Study. Ann Surg Oncol 2016;23(4):1361-70.

18. Rosenberg AM, Friedmann P, Del Rivero J, Libutti SK, Laird AM. Resection versus expectant management of small incidentally discovered nonfunctional pancreatic neuroendocrine tumors. Surgery 2016;159(1):302-10.

19. Partelli S, Cirocchi R, Crippa S, Cardinali L, Fendrich V, Bartsch DK, et al. Systematic review of active surveillance versus surgical management of asymptomatic small non-functioning pancreatic neuroendocrine neoplasms. Br J Surg 2016.

20. Yao JC, Hassan M, Phan A, Dagohoy C, Leary C, Mares JE, et al. One hundred years after "carcinoid": epidemiology of and prognostic factors for neuroendocrine tumors in 35,825 cases in the United States. J Clin Oncol 2008;26(18):3063-72.

21. Yadegarfar G, Friend L, Jones L, Plum LM, Ardill J, Taal B, et al. Validation of the EORTC QLQ-GINET21 questionnaire for assessing quality of life of patients with gastrointestinal neuroendocrine tumours. Br J Cancer 2013;108(2):301-10.

22. Zigmond AS, Snaith RP. The hospital anxiety and depression scale. Acta Psychiatr Scand 1983;67(6):361-70.

23. Therasse P, Arbuck SG, Eisenhauer EA, Wanders J, Kaplan RS, Rubinstein L, et al. New guidelines to evaluate the response to treatment in solid tumors. European Organization for Research and Treatment of Cancer, National Cancer Institute of the United States, National Cancer Institute of Canada. J Natl Cancer Inst 2000;92(3):205-16.

**APPENDIX 1:** Study Flowchart

**APPENDIX 2:** Suggested scheme of active surveillance for sporadic asymptomatic NF-PNEN ≤ 2 cm


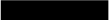


**MANDATORY**

CT scan and/or MRI and/ or ^68^Gallium PET/CT

and/or Octreoscan and/or EUS+FNA and/or histological confirmation

CgA + HADS+ EORTC QLQ-C30 v3 and GI.NET21

Enrolment

**OPTIONAL or MANDATORY if Ki67>2%**

CT scan or MRI

CgA + HADS+ EORTC QLQ-C30 v3 and GI.NET21

6 Months

**MANDATORY**

CT scan or MRI

HADS+ EORTC QLQ-C30 v3 and GI.NET21

12 Months

If sdsd changes

**OPTIONAL or MANDATORY if Ki67>2%**

CT scan or MRI

CgA + HADS+ EORTC QLQ-C30 v3 and GI.NET21

18 Months

**MANDATORY**

CT scan or MRI

HADS + EORTC QLQ-C30 v3 and GI.NET21

24 Months

60 Months

**MANDATORY**

CT scan or MRI

HADS + EORTC QLQ-C30 v3 and GI.NET21

36 Months

**MANDATORY**

CT scan or MRI

HADS + EORTC QLQ-C30 v3 and GI.NET21

*IF NO CHANGES CONTINUE YEARLY*

* Date of surgery does not chance the timing of follow up that starts from the date of enrolment.

^Additional examinations (PET/CT 68Ga, EUS+FNA, CgA) can be performed at treating physician’s discretion

**APPENDIX 3:** Suggested scheme after surgical resection for sporadic asymptomatic NF-PNEN ≤ 2 cm

**MANDATORY**

CT scan or MRI

^68^Gallium PET/CT Or Octreoscan

EUS+FNA

CgA + HADS + EORTC QLQ-C30 v3 and GI.NET21

Or histological confirm of NF-PNEN ≤ 2 cm

UPFRONT SURGERY OR ENROLLMENT*

* Date of surgery does not chance the timing of follow up that starts from the date of enrolment.

**MANDATORY**

CT scan or MRI

CgA + HADS + EORTC QLQ-C30 v3 and GI.NET21

**MANDATORY**

CT scan or MRI

HADS + EORTC QLQ-C30 v3 and GI.NET21

**MANDATORY**

CT scan or MRI

HADS + EORTC QLQ-C30 v3 and GI.NET21

**OPTIONAL or MANDATORY if Ki67>2%**

CT scan or MRI

CgA + HADS + EORTC QLQ-C30 v3 and GI.NET21

**MANDATORY**

CT scan or MRI

HADS + EORTC QLQ-C30 v3 and GI.NET21

**OPTIONAL or MANDATORY if Ki67>2%**

CT scan or MRI

CgA + HADS + EORTC QLQ-C30 v3 and GI.NET21

12 Months

6 Months

60 Months

*IF NO CHANGES CONTINUE YEARLY*

36 Months

24 Months

18 Months

**APPENDIX 4 : Hospital Anxiety and Depression Scale (HADS) available in different languages.**

**Tick the box beside the reply that is closest to how you have been feeling in the past week. Don’t take too long over you replies: your immediate is best.**

| **D** | **A** |  | **D** | **A** |  |
| --- | --- | --- | --- | --- | --- |
|  |  | **I feel tense or 'wound up':** |  |  | **I feel as if I am slowed down:** |
|  | 3 | Most of the time | 3 |  | Nearly all the time |
|  | 2 | A lot of the time | 2 |  | Very often |
|  | 1 | From time to time, occasionally | 1 |  | Sometimes |
|  | 0 | Not at all | 0 |  | Not at all |
|  |  |  |  |  |  |

|  |  | **I still enjoy the things I used to** |  | **I get a sort of frightened feeling like** |
| --- | --- | --- | --- | --- |
|  |  | **enjoy:** |  | **'butterflies' in the stomach:** |
| 0 |  | Definitely as much | 0 | Not at all |
| 1 |  | Not quite so much | 1 | Occasionally |
| 2 |  | Only a little | 2 | Quite Often |
| 3 |  | Hardly at all | 3 | Very Often |
|  |  |  |  |  |

|  |  | **I get a sort of frightened feeling as if** |  |  |  |
| --- | --- | --- | --- | --- | --- |
|  |  | **something awful is about to** |  |  | **I have lost interest in my appearance:** |
|  |  | **happen:** |  |  |  |
|  | 3 | Very definitely and quite badly | 3 |  | Definitely |
|  | 2 | Yes, but not too badly | 2 |  | I don't take as much care as I should |
|  | 1 | A little, but it doesn't worry me | 1 |  | I may not take quite as much care |
|  | 0 | Not at all | 0 |  | I take just as much care as ever |
|  |  |  |  |  |  |

|  |  | **I can laugh and see the funny side** |  | **I feel restless as I have to be on the** |
| --- | --- | --- | --- | --- |
|  |  | **of things:** |  | **move:** |
| 0 |  | As much as I always could | 3 | Very much indeed |
| 1 |  | Not quite so much now | 2 | Quite a lot |
| 2 |  | Definitely not so much now | 1 | Not very much |
| 3 |  | Not at all | 0 | Not at all |

|  |  | **Worrying thoughts go through my** |  |  | **I look forward with enjoyment to** |
| --- | --- | --- | --- | --- | --- |
|  |  | **mind:** |  |  | **things:** |
|  | 3 | A great deal of the time | 0 |  | As much as I ever did |
|  | 2 | A lot of the time | 1 |  | Rather less than I used to |
|  | 1 | From time to time, but not too often | 2 |  | Definitely less than I used to |
|  | 0 | Only occasionally | 3 |  | Hardly at all |
|  |  |  |  |  |  |

|  |  | **I feel cheerful:** |  | **I get sudden feelings of panic:** |
| --- | --- | --- | --- | --- |
| 3 |  | Not at all | 3 | Very often indeed |
| 2 |  | Not often | 2 | Quite often |
| 1 |  | Sometimes | 1 | Not very often |
| 0 |  | Most of the time | 0 | Not at all |
|  |  |  |  |  |

|  |  | **I can sit at ease and feel relaxed:** |  |  | **I can enjoy a good book or radio or TV** |
| --- | --- | --- | --- | --- | --- |
|  |  |  |  |  | **program:** |
|  | 0 | Definitely | 0 |  | Often |
|  | 1 | Usually | 1 |  | Sometimes |
|  | 2 | Not Often | 2 |  | Not often |
|  | 3 | Not at all | 3 |  | Very seldom |

Please check you have answered all the questions

Scoring:

Total score: Depression (D) ___________ Anxiety (A) ______________

0-7 = Normal

8-10 = Borderline abnormal (borderline case)

11-21 = Abnormal (case)


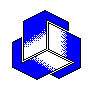
**Appendix 5. EORTC QLQ-C30 Questionnaire (version 3.0)**

**EORTC QLQ-C30 (version 3)**

We are interested in some things about you and your health. Please answer all of the questions yourself by circling the number that best applies to you. There are no "right" or "wrong" answers. The information that you provide will remain strictly confidential.

_____________________________________________________________________________________

**Not at A Quite Very**

**All Little a Bit Much**

1. Do you have any trouble doing strenuous activities,

like carrying a heavy shopping bag or a suitcase? 1 2 3 4

2. Do you have any trouble taking a long walk? 1 2 3 4

3. Do you have any trouble taking a short walk outside of the house? 1 2 3 4

4. Do you need to stay in bed or a chair during the day? 1 2 3 4

5. Do you need help with eating, dressing, washing

yourself or using the toilet? 1 2 3 4

**During the past week: Not at A Quite Very**

**All Little a Bit Much**

6. Were you limited in doing either your work or other daily activities? 1 2 3 4

7. Were you limited in pursuing your hobbies or other

leisure time activities? 1 2 3 4

8. Were you short of breath? 1 2 3 4

9. Have you had pain? 1 2 3 4

10. Did you need to rest? 1 2 3 4

11. Have you had trouble sleeping? 1 2 3 4

12. Have you felt weak? 1 2 3 4

13. Have you lacked appetite? 1 2 3 4

14. Have you felt nauseated? 1 2 3 4

15. Have you vomited? 1 2 3 4

16. Have you been constipated? 1 2 3 4

**During the past week: Not at A Quite Very**

**All Little a Bit Much**

17. Have you had diarrhea? 1 2 3 4

18. Were you tired? 1 2 3 4

19. Did pain interfere with your daily activities? 1 2 3 4

20. Have you had difficulty in concentrating on things,

like reading a newspaper or watching television? 1 2 3 4

21. Did you feel tense? 1 2 3 4

22. Did you worry? 1 2 3 4

23. Did you feel irritable? 1 2 3 4

24. Did you feel depressed? 1 2 3 4

25. Have you had difficulty remembering things? 1 2 3 4

26. Has your physical condition or medical treatment

interfered with your family life? 1 2 3 4

27. Has your physical condition or medical treatment

interfered with your social activities? 1 2 3 4

28. Has your physical condition or medical treatment

caused you financial difficulties? 1 2 3 4

**For the following questions please circle the number between 1 and 7 that
best applies to you**

29. How would you rate your overall health during the past week?

1 2 3 4 5 6 7

Very poor Excellent

30. How would you rate your overall quality of life during the past week?

1 2 3 4 5 6 7

Very poor Excellent

© Copyright 1995 EORTC Quality of Life Group. All rights reserved. Version 3.0

**APPENDIX 6: EORTC QLQ-GI.NET21 Module**


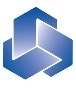
**EORTC QLQ – GI.NET21**

Patients sometimes report that they have the following symptoms or problems. Please indicate the extent to which you have experienced these symptoms or problems during the past week. Please answer by circling the number that best applies to you.

**During the past week: Not A Quite Very**

**at all little a bit much**

31. Did you have hot flushes? 1 2 3 4

32. Have you noticed or been told by others that you looked flushed/red? 1 2 3 4

33. Did you have night sweats? 1 2 3 4

34. Did you have abdominal discomfort? 1 2 3 4

35. Did you have a bloated feeling in your abdomen? 1 2 3 4

36. Have you had a problem with passing wind/gas/flatulence? 1 2 3 4

37. Have you had acid indigestion or heartburn? 1 2 3 4

38. Have you had difficulties with eating? 1 2 3 4

39. Have you had side-effects from your treatment?
*(If you are not on treatment please circle N/A)* N/A 1 2 3 4

40. Have you had a problem from repeated injections?
*(If not having injections please circle N/A)* N/A 1 2 3 4

41. Were you worried about the tumour recurring in other areas of the body? 1 2 3 4

42. Were you concerned about disruption of home life? 1 2 3 4

43. Have you worried about your health in the future? 1 2 3 4

44. How distressing has your illness or treatment been to those close to you? 1 2 3 4

45. Has weight loss been a problem for you? 1 2 3 4

46. Has weight gain been a problem for you? 1 2 3 4

47. Did you worry about the results of your tests?
*(If you have not had tests please circle N/A)* N/A 1 2 3 4

48. Have you had aches or pains in your muscles or bones? 1 2 3 4

49. Did you have any limitations in your ability to travel? 1 2 3 4

**During the past four weeks:**

50. Have you had problems receiving adequate information
about your disease and treatment? 1 2 3 4

51. Has the disease or treatment affected your sex life (for the worse)?
*(If not applicable please circle N/A)* N/A 1 2 3 4
