# Supplementary material for: Management of asymptomatic sporadic non-functioning pancreatic neuroendocrine neoplasms no larger than 2 cm: interim analysis of prospective ASPEN trial
Source: Br J Surg. 2022 Aug 20;109(12):1186–90. doi: 10.1093/bjs/znac267 (PMC10364756; doi:10.1093/bjs/znac267)
Supplement: znac267_Supplementary_Data [file znac267_supplementary_data.zip › Supplementary_Tables.docx]

**Supplementary Tables**

**Table S1**. Univariate and Multivariable analyses of factors associated with surgical management

|  | **Univariate analysis** | | **Multivariable analysis** | |
| --- | --- | --- | --- | --- |
| **Characteristic** | **OR (95% CI)** | ***P* value** | **OR (95% CI)** | ***P* value** |
| **Gender** |  |  |  |  |
| Female | 1 |  |  |  |
| Male | 1.2 (0.7-1.8) | 0.530 |  |  |
| **Age,** years |  |  |  |  |
| ≤64 | 1 |  | 1 |  |
| >64 | 0.4 (0.3-0.7) | <0.001 | 0.4 (0.22-0.6) | <0.001 |
| **BMI,** Kg/m^2^ |  |  |  |  |
| ≤25 | 1 |  | 1 |  |
| >25 | 0.6 (0.4-0.99) | 0.053 | 0.7 (0.5-1.2) | 0.223 |
| **Diabetes**  No  Yes | 1  0.7 (0.6-1.3) | 0.270 |  |  |
| **Radiological tumor size**^b^, mm |  |  |  |  |
| ≤10 | 1 |  | 1 |  |
| >10 | 1.8 (1.05-3.2) | 0.034 | 1.9 (1.1-3.5) | 0.030 |
| **MPD**, mm  ≤1  >1≤3  >3 | 1  1.0 (0.6-1.8)  3.8 (1.9-7.5) | 0.869  <0.001 | 1  0.9 (0.5-1.7)  3.4 (1.7-7.1) | 0.832  0.001 |
| **Site** |  |  |  |  |
| Body/tail | 1 |  |  |  |
| Head | 0.70 (0.4-1.1) | 0.146 |  |  |
| **Surgical Center**  No  Yes | 1  2.0 (1.2-3.4) | 0.007 | 1  2.0 (1.2-3.5) | 0.012 |
| **HADS score** |  |  |  |  |
| ≤6 | 1 |  |  |  |
| >6≤12 | 1.5 (0.8-2.6) | 0.195 |  |  |
| >12 | 1.5 (0.9-2.7) | 0.149 |  |  |
| **HADS-Anxiety score** ^d^ |  |  |  |  |
| ≤3 | 1 |  | 1 |  |
| **>**4≤6 | 1.8 (1.01-3.1) | 0.050 | 2.0 (1.1-3.6) | 0.029 |
| >6 | 1.2 (0.7-2.2) | 0.564 | 1.2 (0.6-2.2) | 0.629 |
| **HADS-Depression score** |  |  |  |  |
| ≤2 | 1 |  |  |  |
| **>**3≤4 | 1.1 (0.6-1.99) | 0.785 |  |  |
| >4 | 1.3 (0.7-2.5) | 0.337 |  |  |

Abbreviations: OR, Odds Ratio; BMI, Body Mass Index; MPD, Main Pancreatic Duct; HADS, Hospital Anxiety and Depression Scale

^a^p values were calculated using *t* test, Wilcoxon Mann-Whitney test, or Pearson χ^2^ test as appropriate.

^b^Maximum size measured at radiological imaging or endoscopic ultrasound

^C^Evaluated on FNA/B specimen. OR calculated on 217 patients with FNA positive and evaluable Ki-67 value

^d^Categorized by tertiles of HADS score distribution

**Table S2.** Surgical Outcomes

| **Characteristic** | **N= 94 (%)** |
| --- | --- |
| **Type of surgical procedure** |  |
| Pancreaticoduodenectomy | 19 (20) |
| Central pancreatomy | 2 (2) |
| Distal pancreatomy | 56 (60) |
| Enucleation | 17 (18) |
| **Surgical approach** |  |
| Laparoscopy | 35 (37) |
| Laparotomy | 42 (45) |
| Robot assisted | 17 (18) |
| **ASA score** |  |
| 0 | 14 (15) |
| I | 19 (20) |
| II | 49 (52) |
| III | 12 (13) |
| **Complication grade**^a^ |  |
| No complications | 64 (68) |
| Grade 1 | 11 (12) |
| Grade 2 | 7 (7) |
| Grade 3 | 8 (9) |
| Grade 4  Grade 5 | 4 (4)  0 (0) |
| **Operative time, minutes, mean (SD)** | 222 (±120.6) |
| **Intraoperative Bleeding, ml, mean (SD)** | 161 (±234.5) |
| **Length of hospital stay, days, mean (SD)** | 9.5 (±6.6) |

Abbreviations: ASA, American Society of Anesthesiologists; POPF, Postoperative pancreatic fistula;

^a^Severity of complications was classified according to Dindo et al. ^15^

**Table S3.** Postoperative pathological findings

| **Characteristic** | **N= 94 (%)** |
| --- | --- |
| **Tumor size**^a^**, mm, mean (SD)** | 13 (±4.8) |
| **Tumor grading** |  |
| G1 | 73 (78) |
| G2 | 20 (21) |
| G3* | 1 (1) |
| **Ki-67, %, mean (SD)** | 3 (±4) |
| **Perineural Invasion** |  |
| No | 85 (90) |
| Yes | 9 (10) |
| **Microvascular invasion** |  |
| No | 87 (93) |
| Yes | 7 (7) |
| **pT status** |  |
| T1 | 89 (95) |
| T2 | 5 (5) |
| **pN status** |  |
| N0 | 76 (81) |
| N1 | 5 (5) |
| Nx | 13 (14) |
| **R** |  |
| R0 | 94 (100) |
| **M status** |  |
| M0 | 90 (96) |
| M1 | 4 (4) |
| **Aggressive Features**^d^  No  Yes | 75 (80)  19 (20) |

^a^Maximum size measured by pathologist

^d^Including at least one of the following: Ki67 >20%, perineural invasion, microvascular invasion, nodal metastases, distant metastases

*Well-differentiated NET-G3
