# Supplementary figures and images for: Management of asymptomatic sporadic non-functioning pancreatic neuroendocrine neoplasms no larger than 2 cm: interim analysis of prospective ASPEN trial
Source: Br J Surg. 2022 Aug 20;109(12):1186–90. doi: 10.1093/bjs/znac267 (PMC10364756; doi:10.1093/bjs/znac267)

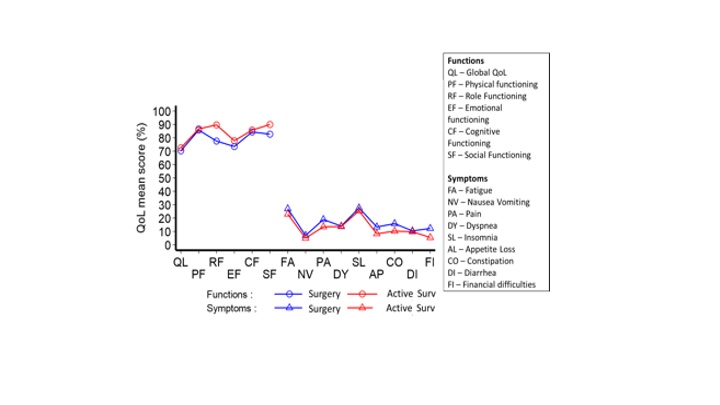

Supplement: znac267_Supplementary_Data [file znac267_supplementary_data.zip › Supplementary_Figure_1.jpg]
